# Quantification of Hordeins by ELISA: The Correct Standard Makes a Magnitude of Difference

**DOI:** 10.1371/journal.pone.0056456

**Published:** 2013-02-28

**Authors:** Gregory J. Tanner, Malcolm J. Blundell, Michelle L. Colgrave, Crispin A. Howitt

**Affiliations:** 1 Commonwealth Scientific and Industrial Research Organisation Plant Industry, Canberra, Australian Capital Territory, Australia; 2 Commonwealth Scientific and Industrial Research Organisation Food Futures Flagship, North Ryde, New South Wales, Australia; 3 Commonwealth Scientific and Industrial Research Organisation Animal, Food and Health Sciences, St Lucia, Queensland, Australia; Tsinghua University, China

## Abstract

**Background:**

Coeliacs require a life-long gluten-free diet supported by accurate measurement of gluten (hordein) in gluten-free food. The gluten-free food industry, with a value in excess of $6 billion in 2011, currently depends on two ELISA protocols calibrated against standards that may not be representative of the sample being assayed.

**Aim:**

The factors affecting the accuracy of ELISA analysis of hordeins in beer were examined.

**Results:**

A simple alcohol-dithiothreitol extraction protocol successfully extracts the majority of hordeins from barley flour and malt. Primary hordein standards were purified by FPLC. ELISA detected different classes of purified hordeins with vastly different sensitivity. The dissociation constant (Kd) for a given ELISA reaction with different hordeins varied by three orders of magnitude. The Kd of the same hordein determined by ELISA using different antibodies varied by up to two orders of magnitude. The choice of either ELISA kit or hordein standard may bias the results and confound interpretation.

**Conclusions:**

Accurate determination of hordein requires that the hordein standard used to calibrate the ELISA reaction be identical in composition to the hordeins present in the test substance. In practice it is not feasible to isolate a representative hordein standard from each test food. We suggest that mass spectrometry is more reliable than ELISA, as ELISA enumerates only the concentration of particular amino-acid epitopes which may vary between different hordeins and may not be related to the absolute hordein concentration. MS quantification is undertaken using peptides that are specific and unique enabling the quantification of individual hordein isoforms.

## Introduction

Coeliac disease (CD) occurs in 1% of most populations worldwide, numbering an estimated 70 million people globally. CD currently requires lifelong dietary exclusion of gluten proteins in wheat (gliadin and glutenins), barley (hordeins), rye (avenins) and in some individuals, oats (secalins). Untreated coeliacs face a raft of adverse health outcomes including low bone mineral density and increased intestinal malignancy [1]. In addition to coeliacs there is a larger group who are intolerant to gluten containing foods. Unlike coeliac disease, the molecular basis of gluten intolerance is largely unreported [2]. A smaller group of people suffer from a rapid onset, IgE mediated, anaphylactic allergy to gluten which can be life threatening [3]. All of these groups require a lifelong gluten-free diet.

The WHO standard for gluten-free foods, adopted by the Codex Alimentarius [4] in 2008 requires that food prepared from wheat, barley, rye and oats must contain less than 20 mg/kg (µg/ml, parts per million, ppm) gluten to be labelled “gluten-free”. Most international jurisdictions are adopting similar regulations, however in Australia, FSANZ [5] adds the additional caveat that gluten-free food cannot be made from cereals containing gluten or from foods with any detectable gluten level. This “catch-all” clause was once sensible; however with newer detection techniques such as MS it needs to be revised. The global gluten-free food industry, with a value in excess of $6 billion in 2011, is predicted to grow by US$1.2 billion over the next five years [6]. This expanding economic market depends on two approved antibodies for validation of the gluten-free status of food products. These two ELISA sandwich kits have been ring-tested, and accepted by the Food and Agriculture Organisation of the United Nations (FAO) for measuring gluten concentrations in flour and food. The kits are based on one of two antibodies. The Mendez R5 antibody (RidaScreen) [7], uses the mouse monoclonal R5 antibody raised against rye secalins by Mendez [8] and which recognises QQPFP, QQQFP, LQPFP and QLPFP epitopes. The second FAO accepted sandwich ELISA kit is based on the Skerritt antibody (ELISA Systems & Tepnal [9]) that uses the mouse monoclonal antibody MAb41201, which is functionally equivalent to M12224 raised to wheat ω-gliadins (Skerritt [10]) and recognises the epitopes PQPQPFPQE and PQQPPFPEES [11].

The development of standardised gluten sources [12] represents significant progress towards the accurate determination of gluten levels in flour samples, however, this is confounded because there is not a single gluten protein in flour, but many hundred different gluten proteins which vary from sample to sample. To date there are no suitable hordein standards for beer, however the detection of hordeins in malt and beer by competitive ELISA has been reported [13], [14], [15].

Second generation sandwich ELISA methods have been developed using antibodies raised against specific immuno-dominant peptides involved in the biological response of coeliac disease, e.g. G12 and A1 monoclonal antibodies have been raised against the toxic 33-mer of α-gliadin [16], [17], [18]. This peptide (LQLQPFPQPQLPYPQPQLPYPQPQLPYPQPQPF) encodes six partially overlapping T-cell epitopes and has potent T-cell stimulatory properties [19]. It also contains the so-called p56–75 peptide (LQLQPFPQPQLPYPQPQLPY) that has been identified as the dominant immuno-reactive peptide for coeliacs. This peptide accounts for the bulk of coeliac toxicity in all gluten proteins [20]. Homologues to the 33mer peptide are also found in barley, oats and rye. Development of anti-33-mer antibodies represent a development over Mendez and Skerritt first generation antibodies, raised against general protein fractions from rye secalins [8] or wheat ω-gliadins [10].

Another peptide based approach is possible. Three highly immunogenic peptides, derived from α-gliadin (ELQPFPQPELPYPQPQ), ω-gliadin/C-hordein (EQPFPQPEQPFPWQP), and B-hordein (EPEQPIPEQPQPYPQQ), account for 90% of the coeliac-specific immuno-response elicited by the full complement of wheat, barley and rye proteins. These three peptides provide the basis for a vaccination approach to reduce CD [21]. This also suggests that the use of antibodies raised against these key peptides may also provide a direct measure of the level immuno-reactive epitopes present in a grain sample.

The first step in activating gluten peptides for the coeliac cascade is the deamination of specific glutamine residues in partially hydrolysed gluten peptides by human tissue transglutaminase (tTG) [22]. The resultant glutamate residues bind to HLA-DQ2 or -DQ8 receptors on lymphocyte antigen presenting cells. This step stimulates the clonal propagation of specific T-cells which are targeted to the intestine and initiate the ultimate destruction of intestinal villi. A novel approach has recently used the enzyme specificity of tTG, to deaminate peptides carrying biologically important glutamines, followed by labelling of the resultant glutamic acids. Twenty two peptides from wheat, barley and rye were specifically labelled and identified by HPLC-mass spectrometry (MS) [23]. Surprisingly, one of these peptides, QPQQPLPQPQQPF was present in wheat, barley and rye and forms a logical basis for a peptide to quantify gluten in these grains [24].

Determination of hordein in beer is more difficult. In practice, beers are often produced from a blend of barley varieties, so it is not always possible to identify appropriate controls. In addition hordeins may be modified by hydrolysis, glycation (the non-enzymatic, covalent addition of a sugar) and glycosylation (enzymatic covalent addition of a sugar). Hydrolysis of peptides, so that only one epitope is present in a given peptide, may confound sandwich ELISA systems which require two epitopes, one to bind the antibody coated on the ELISA plate, and one to bind the quantifier – usually a HRP labelled antibody conjugate added in solution.

Low levels of hordein in beer have been detected by sandwich ELISA [13], [14], [15], [25] and improvements for the determination of hydrolysed hordeins developed [26]. Measurement of immuno-reactive gluten peptides in beer using the newer G12 antibody have also been reported [27] and recently reviewed [28]. The differential immuno-reactivity of commercial immunoassay kits has been studied. It was shown that antibodies from the R-Biopharm, Morinaga and Romer laboratories reacted strongly with gliadin, whereas those from BioKits, ALLER-TEK and ELISA Systems reacted strongly with glutenin [29]. Several improvements to existing ELISA technology have been proposed. The use of a novel chicken egg yolk anti-gliadin immunoglobulin Y as capturing antibody and monoclonal anti-gliadin IgG (HYB-314) antibody as detecting antibody for improved detection of gliadins has recently been reported [30]. The use of chymotryptic proteolysis has recently been shown to selectively cleave and reduce cross reactive epitopes (false positives) while retaining the target epitope in ELISA analysis of a novel ‘S’-type low molecular weight glutenin subunit [31].

Barley is a diploid and unlike the situation in bread wheat, the genetics of hordeins are relatively straightforward. There are four protein families of hordeins: B-, C-, D- and γ-hordeins, with the B- and C-hordeins together accounting for over 90% of barley hordeins [32]. Isolation of hordein double-null barley lines from hybrids of Risø 56 and Risø 1508 has produced a line which does not accumulate B- or C-hordein and only has 3% of wild type hordein along with a 20-fold reduction in reactivity in T-cell assays [33]. This Ultra-Low- Gluten (ULG 2.0) barley line, along with the parents, contain known but varied hordein compositions and provide a suite of grains suitable for investigating the effect of grain hordein composition on the hordein contents of flour, malt, wort and beer.

We show here that ELISA analysis calibrated with a single prolamin standard produces a relative indication of the hordein level at best. Depending on the standard used, the quantification may over- or under-estimate by several orders of magnitude. Indeed there is a general assumption with publications reporting ELISA analysis of gluten (hordein) that the use of a gliadin standard is appropriate. We show this is incorrect and can lead to serious over- or under-estimation of the hordein content. The former situation, a false positive, impacts on the market opportunities for GF food manufacturers, and reduces the range of food available to coeliacs – a diet which is costly [34], low fibre and minerals, and high in sugar [35], [36]. The latter situation, a false negative, exposes coeliacs to food which is unsuitable and which may induce adverse clinical reactions. In the long run this may contribute to a raft of adverse health outcomes for coeliacs including increased rates of intestinal malignancy such as a 10-fold increased risk of intestinal cancer, a 3- to 6-fold increase in the risk of non-Hodgkin lymphoma and a 28-fold increased risk of intestinal T-cell lymphoma [37].

In an accompanying paper (Tanner et al, this issue, this journal) we demonstrate that ELISA results often do not reflect the hordein content of beer as determined by MS. We suggest that mass spectrometry is more reliable than ELISA, as ELISA enumerates only the concentration of particular amino-acid epitopes which may vary between different hordeins and may not be related to the absolute hordein concentration. MS quantification is undertaken using peptides that are specific and unique enabling the quantification of individual hordein isoforms. We demonstrate that the density of ELISA epitopes varies between different hordein proteins. We demonstrate that the strength of the antibody-protein interaction (measured by the Kd) also varies between different hordein proteins for a particular antibody. The Kd of particular hordeins varies between different antibodies. We suggest that approaches using MS to detect, identify and quantify biologically significant peptides offer the most promising approach to quantitate gluten (hordein) in beverages and foods.

## Results

### Validation of Commercial Antibody by Western Blotting

**Figure 1:**
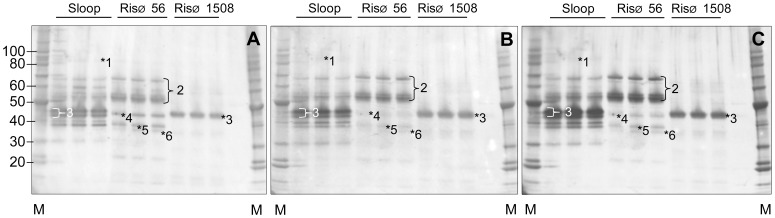
Validation of western blots. Total hordeins (2 µg) from Sloop, Risø 56, or Risø 1508 were subject to 1D SDS-PAGE, blotted and visualised in order with A: anti-gliadin-HRP conjugate (Sigma), then B: rabbit anti-hordein, then C: mouse monoclonal antibody MAB 12224 and counter stained with appropriate HRP-antibody as described and compared to standard proteins (M; Benchmark Protein Ladder, Invitrogen). The location of known hordeins was confirmed by protein sequencing of replicate gels [33]: 1, D-hordeins; 2, C-hordeins; 3, B-hordein; 4, γ-1-hordein; 5, γ-2-hordein; 6, γ-3-hordein.

**Figure 2:**
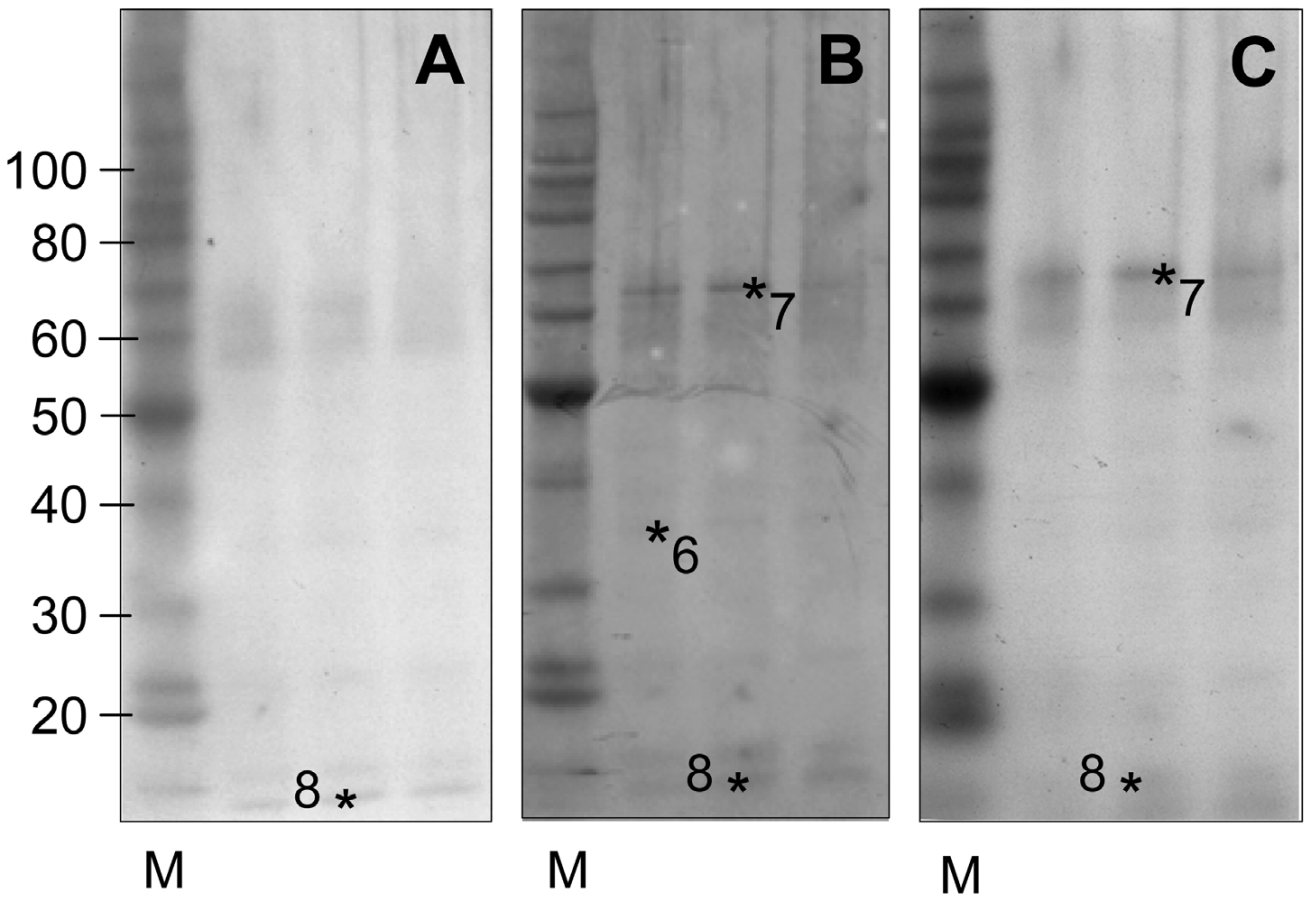
The lack of hordeins in extracts of the hordein double-null line ULG 2.0. Total hordeins (2 µg) from ULG 2.0 were subject to 1D SDS-PAGE, blotted and visualised as above and compared to standard proteins (M; Benchmark Protein Ladder, Invitrogen). The location of known proteins was confirmed by protein sequencing of replicate gels [33]: 6, γ-3-hordein; 7, β-glucosidase; 8, LTP-1.

The composition of alcohol soluble extracts from the wild type, single and double hordein-null grains were analysed by western blot with three anti-hordein antibodies (Fig. 1 & 2). The banding pattern of cv Sloop, Risø 56 and Risø 1508 showed the anti-gliadin-HRP conjugate (Sigma) detected all hordein families: D-hordein was observed as a faint band at ∼90 kDa in Sloop (not detected in ULG 2.0); C-hordeins were observed at 55 and 70 kDa in Risø 56; B-hordeins were observed at 43–45 kDa in Sloop and Risø 1508; and the three γ-hordeins, γ-1, γ-2 and γ-3, were observed at 45, 38 and 35 kDa respectively in Risø 56 (Fig. 1A, 1–6 respectively). The γ-hordeins are normally obscured by co-migrating B-hordeins which do not accumulate in Risø 56. These blots were stripped of antibodies, re-blocked and re-probed with alternate anti-hordein antibodies. The rabbit and mouse antibodies also reacted with all hordein families (Fig. 1B & C). However, the reaction with γ-hordein bands was less intense with both alternate antibodies. A faint western band corresponding to γ-hordein-3 was seen (Fig. 2B, 6). It was noted that both antibodies also reacted with a band corresponding to β-glucosidase at 65 kDa and LTP1 at approximately 10 kDa in extracts of ULG 2.0 (Fig. 2∶7 and 8 respectively). The lower level of hordein in ULG 2.0 results in a much lower level of signal from the respective western blot (Fig. 2) compared to hordein wild-type or single-null (Fig. 1).

**Figure 3:**
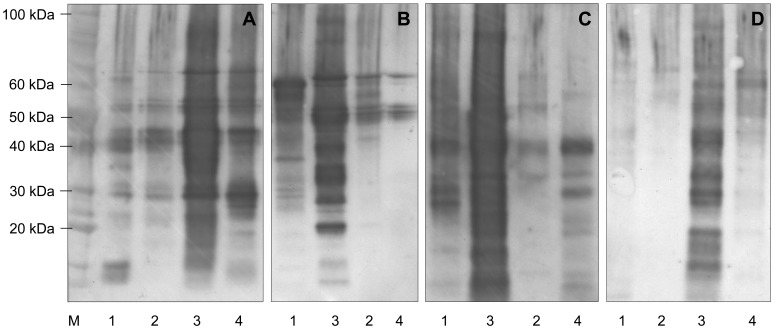
Validation of hordein extraction methods. Half seed extracts were sequentially extracted from cv Sloop (A), Risø 56 (B), Risø 1508 (C), ULG 2.0 (D) in water (1), then 0.5 M NaCl (2), then IPA/DTT (3), then Urea/DTT (4), and 20 µg of total protein from each extract was subject to SDS-PAGE, blotted, interrogated with commercial anti-gliadin-HRP (Sigma) and compared to standard proteins (M; Benchmark Protein Ladder, Invitrogen). Note lanes 2 and 3 are reversed in panels B and C. This blot was deliberately overloaded with protein, to maximise detection of hordeins in the aqueous and salt fractions, causing over exposure of lane 3. When loaded at a lower protein loading of 2 µg per lane a well resolved banding pattern was seen as in Fig. 1A.

**Figure 4:**
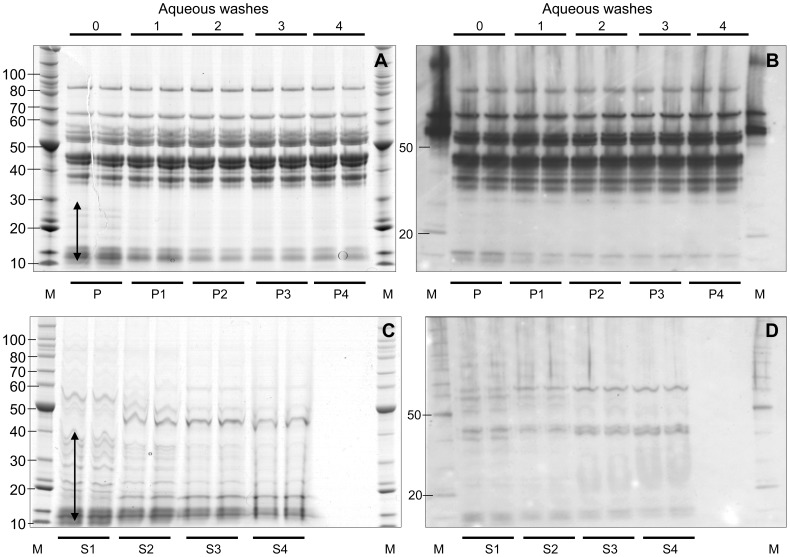
Hordeins do not bleed off in aqueous washes. Duplicate 50 mg aliquots of Sloop flour were extracted in 1 mL of 50% IPA containing 1% (w/v) DTT (IPA/DTT) and an aliquot containing 20 µg protein dried in a SpeedyVac, dissolved in Urea/SDS and resolved on duplicate SDS-PAGE gels (lanes P) which were either stained in colloidal Coomassie Blue (A and C) or blotted to nitrocellulose (iBLOT Promega), blocked in PBST containing 5% skim milk powder, 1% (w/v) Tween overnight at 4°C. The blot was exposed to anti-gliadin-HRP (Sigma) diluted at 1/2000 for 30 min, then washed in PBST, and the signal produced by ECL reagent (Amersham) exposed to Hyperfilm (Amersham) (B and D). Similarly, 50 mg of duplicate flour samples were first washed in 1 mL of water, and centrifuged to give a supernatant and a pellet, which was dissolved in IPA/DTT. Aliquots containing 20 µg protein from both pellet (P1) and supernatant (S1) were dried, redissolved and subject to SDS-PAGE as above. The process was repeated with additional aqueous washing steps to produce supernatants S2–4 and pellets P2–4. Low molecular weight proteins were removed from pellets (A: arrowed) and extracted into supernatants (C: arrowed) after 2 washes. Proteins were calibrated with Benchmark protein ladder (St; Invitrogen).

**Figure 5:**
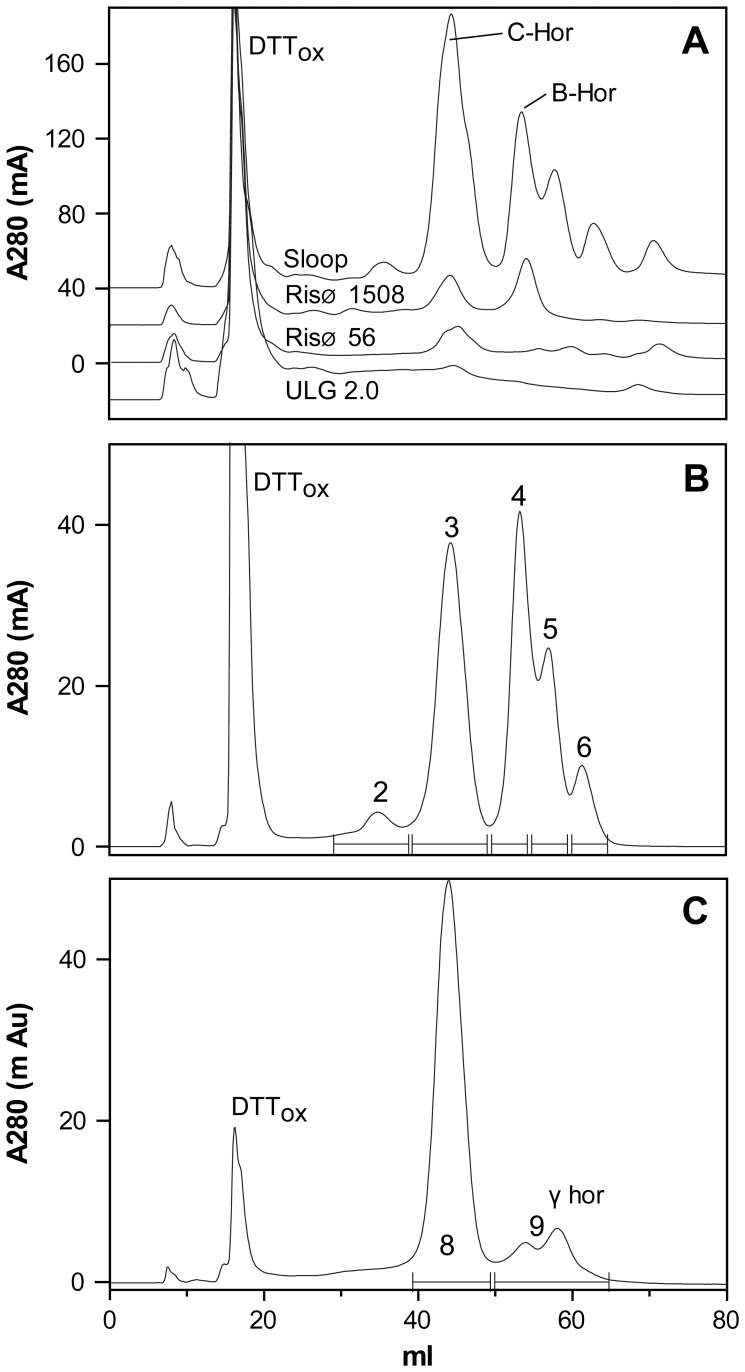
Isolation of hordein fractions by FPLC. A: The eluant A280 from a 1 mL injection of Urea/DTT extract from cv Sloop, Risø 1508, Risø 56 or ULG 2.0 is shown with individual curves offset for clarity. The identity of peaks containing oxidised DTT (DTT_ox_), B- (B-Hor), C- (C-Hor), and γ (γ-Hor)-hordeins of cv Sloop are shown. The eluate from 20 mL to 80 mL was pooled and lyophilised for total hordeins from each line. B & C: Fractions enriched for C-hordeins from cv Sloop (B, peak 3) and Risø 56 (C, peak 8); and γ-hordeins from Risø 56 (C, peaks at 9) were isolated by pooling the indicated eluate (–). Peak 2 did not contain any protein. Peaks 4, 5, 6 were not analysed.

**Figure 6:**
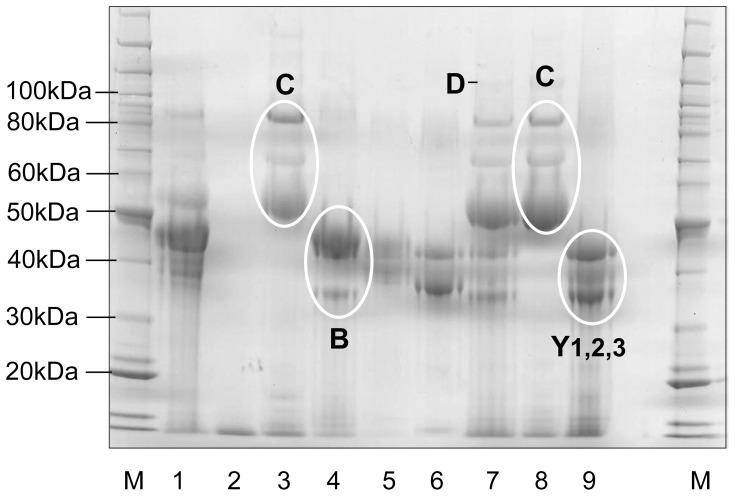
SDS-PAGE of hordein fractions corresponding to the protein peaks in **Fig 9A, B, and C** were pooled as indicated, re-chromatographed, lyophilised, and 20 µg of each fraction examined by SDS-PAGE and stained with Coomassie G250. Lane 1, total hordein from cv Sloop; 2, did not contain any protein; 3, C-hordein from cv Sloop; 4, B-hordein from cv Sloop; 5, γ-1-, -2-hordeins from cv Sloop; 6, γ-1-, -2-, -3-hordeins from cv Sloop; 7, total hordein from Risø 56; 8, C-hordein from Risø 56; 9, γ-1, -2, -3 hordeins from Risø 56. A faint D-hordein band was seen in lane 7. Proteins were compared to standard proteins (M; Benchmark Protein Ladder, Invitrogen).

The similarity in the western blots using three different anti-hordein antibodies validates the use of the Sigma anti-gliadin antibody as a general reagent to detect hordeins. This antibody-reporter conjugate is convenient to use as it is commercially available, sensitive and detects all classes of hordein.

### Validation of Hordein Extraction Methods

Many methods for extraction and solubilisation of gluten or hordeins have been published, but most approximate extraction with a 50% (v/v) alcohol based solvent in the presence of reducing agents such as DTT (recently reviewed [38], [39]). A strong disulfide reducing agent such as DTT or mercaptoethanol is required to cleave inter- and intra-molecular disulfide linkages which may covalently link proteins and otherwise form insoluble protein complexes [38], [40]. Extraction of gluten complexes with chaotropes such as guanidine-HCl or urea have also been reported [41]. In this case the initial extract must be diluted considerably to prevent denaturation of antibodies used in subsequent ELISA analysis.

We verified the efficacy of a simple IPA/DTT solvent extraction by western blots of total protein from half seed extracts of cv Sloop, Risø 56, Risø 1508 and ULG 2.0 interrogated with the Sigma commercial anti-gluten polyclonal antibody (Fig. 3). Very little antibody reactive protein was extracted by water or 0.5 M NaCl. In some cases, traces of the dominant bands seen in the IPA/DTT lane were also present in the water, NaCl and urea extracts. However, in all grains, the majority of the gluten antibody reactive proteins were extracted by IPA/DTT. Alternatively, lyophilised preparations may be dissolved with Urea/DTT or Urea/SDS solutions; the latter is particularly useful as a solubilisation step prior to SDS-PAGE.

### Aqueous Washing does not Solubilise Hordeins

Hordeins did not “bleed-off” with extended aqueous washes of barley flour (Fig. 4). Western blots of successive duplicate aqueous washed pellets which were then extracted in IPA-DTT give a similar pattern on Coommassie stained gels with no evidence of the dominant proteins decreasing in subsequent pellets (Fig. 4A) and increasing in subsequent supernatants (Fig. 4C). Similarly, hordeins revealed by western blotting also did not decrease in the IPA-DTT soluble pellets (Fig. 4B) and did not increase in the aqueous supernatants (Fig. 4D). The signal from the supernatant (Fig. 4D) was much less intense than from the pellet (Fig. 4B), confirming that hordeins were not soluble in the aqueous supernatant. However, a small amount of low molecular weight protein between 10 and 40 kDa was extracted by aqueous washes and was visualised by additional protein bands in supernatant S1, S2, S3 (Fig. 4C, arrowed) and a depletion of similar bands in pellets P and P1 (Fig. 4A, arrowed). This material did not contain hordeins since it did not react with western blots (Fig. 4B & D). Two aqueous washes were sufficient to remove this water soluble protein that was partially soluble in IPA-DTT.

### Purification of Hordein Standards by Fast Protein Liquid Chromatography (FPLC)

Primary hordein standards were purified by solvent extraction, precipitation, FPLC (Fig. 5) and lyophilisation. Identity of the FPLC peaks in Fig. 5 was established by SDS-PAGE of the lyophilised fractions (Fig. 6) and in-gel protein sequencing [33]. The large FPLC peak eluting at 15 mL corresponded to oxidised DTT (Fig. 5). The A280 elution profile was calibrated with extracts of the hordein wild-type (Fig. 5A: Sloop), hordein single-nulls (Fig. 5A: RisØ 1508 and RisØ 56) and hordein double-null line (Fig. 5A: ULG 2.0). C-hordein eluted first at 45 mL, followed by B-hordein (55 mL) and the γ-hordeins as partially resolved peaks from 55–60 ml (Fig. 5C). Unfortunately, we have not succeeded in purifying D-hordein fractions by FPLC. The small A280 peak at 35 mL (Fig. 5B, peak 2) in extracts of Risø 1508 was not due to protein (Fig. 6, lane 2). Fractions enriched for C-hordeins were isolated from cv Sloop (Fig. 1B, peak 3) and Risø 56 (Fig. 1C, peak 8). Fractions enriched for γ-hordeins were isolated from Risø 56 (Fig. 1C, peaks at 9) by pooling the indicated eluate. C-hordeins (Fig. 6, lanes 3 and 8) were observed at 55, 65 and 80 kDa. The γ-1, -2 and -3 hordeins (Fig. 6, lane 9) were observed at 35, 40 and 45 kDa respectively. These bands are normally masked by overlapping B-hordein bands (Fig. 6, lane 4) which are observed at 45–50 kDa, but which do not accumulate in the B-hordein deletion mutant Risø 56 (Fig. 6, lane 7).

### Optimisation of ELISA Systems Kit

Optimisation of the ELISA assay was performed using ELISA Systems kits which are based on the Skerritt antibody [10] which was used in the preliminary Western blotting experiments (Figs. 1 & 2).

**Figure 7:**
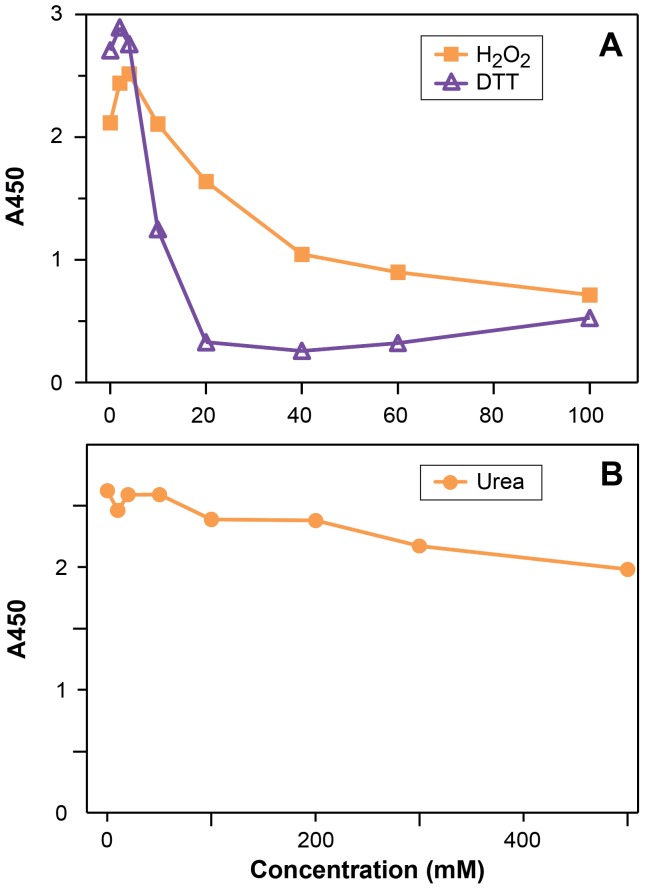
The effect of DTT, hydrogen peroxide and urea on the ELISA response. The response of ELISA Systems sandwich assay containing total Sloop hordein (500 ppb) and either (A) DTT, or H_2_O_2_ or (B) urea, diluted in ED buffer and added to the ELISA wells at the concentration indicated above and processed as described.

Where large volumes of IPA/DTT extract were used, DTT carry-over disrupted antibody binding and reduced the final A450 (Fig. 7A) [42]. Excess DTT of 0.5, 1.0 or 2.0 mM reduced the A450 of 40 ng of Sloop-T hordein by 0, 30% or 69% respectively. Attempts to remove the DTT by precipitating the proteins (2D Clean Up Kit, GE Health Care) were unsatisfactory due to inconsistent re-solubilisation of the precipitate in the aqueous based ELISA Systems diluent. The use of excess H_2_O_2_ (2 mM) restored the A450 of total Sloop hordein, however, concentrations higher than 10 mM resulted in progressive reduction of the A450 signal. An excess of 40 mM H_2_O_2_ reduced the A450 of total Sloop hordein by 50% (Fig. 7A). Excess urea up to 100 mM had no significant effect on the A450 of total Sloop hordein (Fig. 7B).

**Figure 8:**
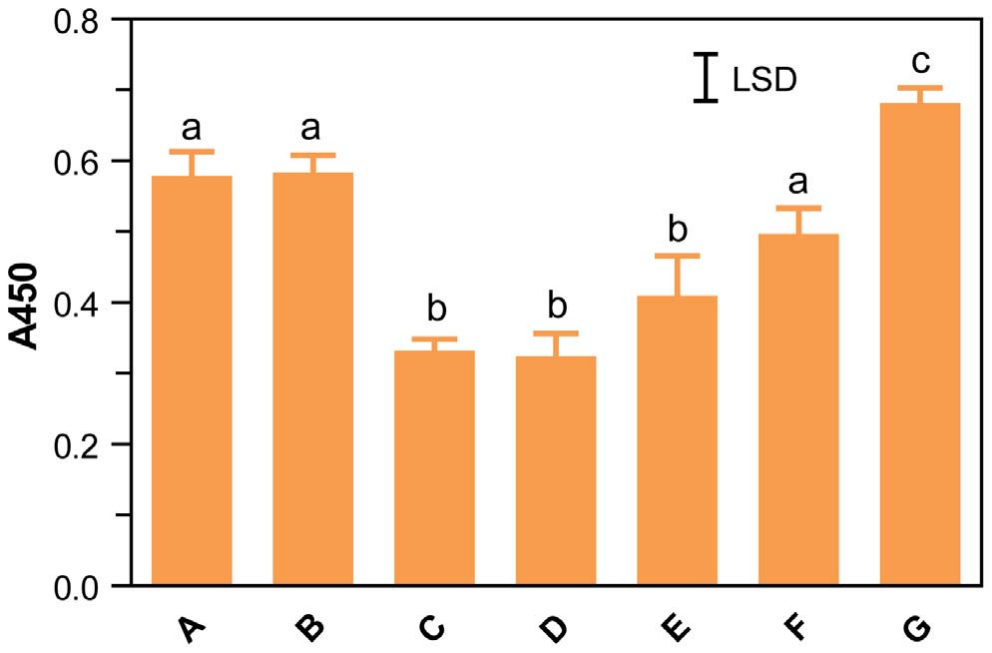
Optimisation of dilution and extraction protocol for ELISA Systems assay. The response of the ELISA Systems sandwich assays to a commercial lager, (Tanner this journal, this issue, Beer 7) extracted with different buffers was measured by adding triplicate samples of commercial beer (100 µL) to 0.9 mL of either: (A & B) dilute ELISA Systems extraction buffer; (C, D, and E) *Ridascreen* extraction cocktail; (F) 60% (v/v) ethanol; or (G) Urea/DTT. The solutions were mixed at either room temperature (A, C, D, F, and G) or 60°C (B, E) for 1 h and an aliquot diluted 1/100 fold with ED buffer (A, B, D, F, G). Solutions C and E were diluted with ten-fold with 80% (v/v) ethanol, and then diluted a further ten-fold with dilute RD buffer and assayed for hordein. Hordeins were measured by adding duplicate 50 µL aliquots to 50 µL of ED buffer in ELISA Systems wells and assayed as described. The mean A450±SE is shown. The maximum concentrations of urea and DTT in assays of solution G were 40 mM and 0.3 mM respectively. The untransformed data were analysed by one-way ANOVA and the LSD is shown. Columns with the same letter were not significantly different (GenStat).

The composition of the initial dilution buffer had a significant effect on the final signal produced by beer in the ELISA Systems assay. An initial 10-fold dilution with Urea/DTT was most successful (Fig. 8; G), followed by initial dilutions with ED buffer (Fig. 8; A, B). Initial dilution with *Ridascreen* extraction cocktail or 60% (v/v) ethanol produced a lower final absorbance (Fig. 8; C, D, E, F). Altering the temperature at which this dilution was performed (at either room temperature or 60°C) did not significantly affect the final absorbance (Fig. 8; A vs B; C vs E). Further dilution of the *Ridascreen* extractions with either RD buffer (Fig. 8; C) or ED buffer (Fig. 8; D) did not increase the final signal. The signal produced following an initial dilution in 60% (v/v) ED buffer or EtOH, followed by dilution with ED buffer were not significantly different (Fig. 8; A vs F). Based on these results, the optimum dilution schedule for beer with the ELISA Systems kit was an initial 1/10 dilution with Urea/DTT, followed by a second dilution of at least 1/100 with ED buffer (Fig. 8; G).

**Figure 9:**
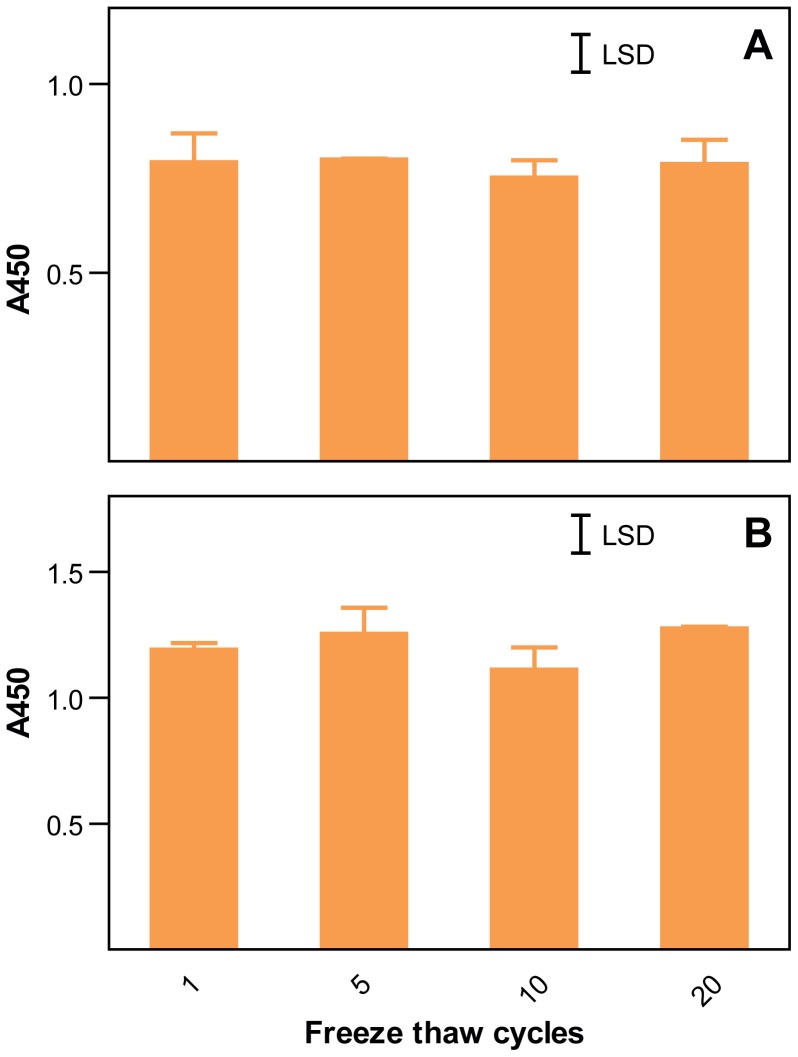
Repeated freeze thaw cycles do not affect the ELISA response. One mL aliquots of either total Sloop hordein (1.0 mg/mL; in a solution containing 8 M urea, 1% (w/v) DTT, 20 mM triethanolamine-HCl adjusted to pH 6 (A) or Sloop beer (B) were repeatedly frozen in liquid nitrogen and thawed in a water bath at RT. Aliquots were retained at 4°C after the indicated freeze-thaw cycle and diluted with ED buffer as required and duplicates analysed for hordeins by an ELISA Systems kit. The mean A450±SE is shown. Error bars are not shown where the S.E. is less than the line thickness. In each experiment the means were not significantly different (P<0.05) by one-way ANOVA (GenStat) and the least significant difference (LSD) is shown. Final concentration of hordein was 1000 ppb. Final dilution of Sloop beer was 1/500.

In order to ensure that sample handling did not affect the results of the ELISA analysis, the beer and hordein standards were subjected to 20 freeze-thaw cycles. No significant loss of reactivity was observed with beer or hordein standard (Fig. 9).

We also assessed the effect of using different polypropylene containers to evaluate possible adsorption of low protein levels. The type of polypropylene container did not significantly affect the final signal produced by final Sloop total hordein concentrations of 1000, 500 or 200 ppb. However, the use of polystyrene plates (BioOne, Frickenhausen, Germany, 96×0.2 mL) for the dilution, reduced the final signal at all of the above hordein dilutions by approximately 50% compared to the polypropylene containers with the ELISA Systems kit. Addition of 1 ppm BSA as a sorbent did not restore the signal. A similar effect was noted with dilute beer solutions. When the commercial lager was diluted in polystyrene wells the final signal at dilutions of 1/1000, 1/2000 or 1/5000 were 74%, 45% and 37% respectively of the average level observed for all the above polypropylene containers with the ELISA Systems kit. As noted previously, the addition of 1 ppm BSA did not restore the signal produced by beers.

**Figure 10:**
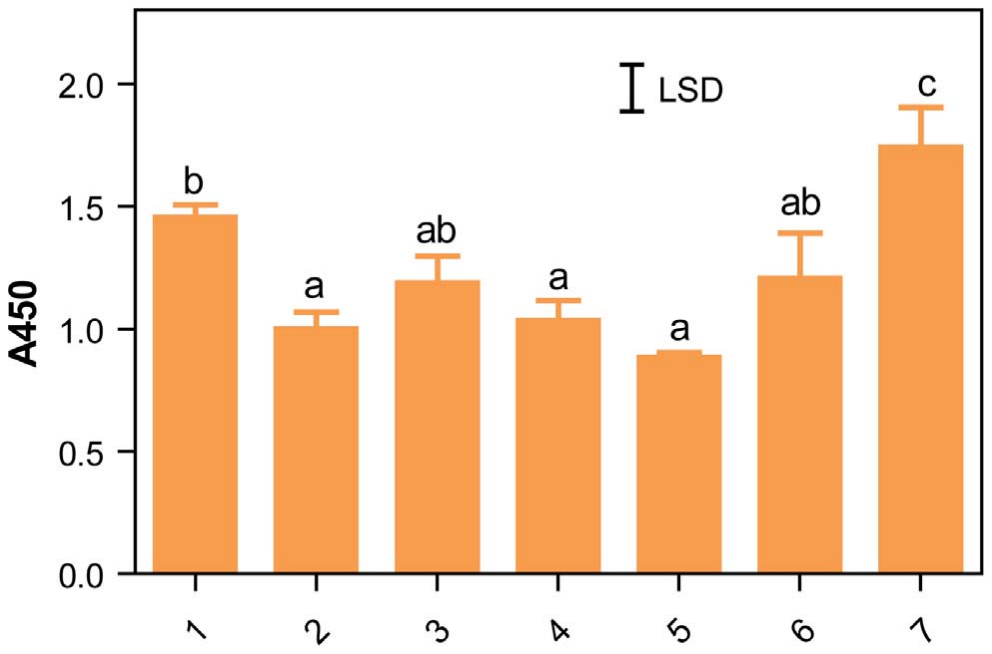
The hordein content of wild type flours varies with variety and environment. The response of ELISA Systems anti-gluten sandwich assays to triplicate 10 ng alcohol soluble protein from different wild type flours. Mean A450±SE is shown for triplicate extracts for cv Sloop grown at CSIRO Ginninderra Experiment Station, Canberra, 1 or Yanco Agricultural Institute (NSW Dept. of Agriculture, Yanco, NSW), 2; cv Himalaya, 3; cv Bomi, 4; cv Carlsberg II, 5; cv Hindmarsh, 6; and cv Commander, 7, all grown at CSIRO Ginninderra Experiment Station, Canberra. Total protein was dissolved in Urea/DTT, the protein content measured, diluted 1/500 with ESD, and duplicate aliquots containing 10 ng of protein in approximately 2 µL added to ELISA wells containing a final volume of 100 µL ESD and the sandwich assay carried out. The untransformed data were analysed by one-way ANOVA and the LSD is shown. Columns with the same letter were not significantly different (GenStat).

The hordeins in 10 ng of alcohol soluble protein from wild type flours varied significantly (Fig. 10). Extracts of cv Commander were the most reactive, followed by extracts of cv Sloop. Extracts of cv’s Himalaya, Bomi, Carlsberg II, and Hindmarsh were not significantly different from each other. Extracts of cv Sloop grown at two different locations also varied significantly. The hordein concentration in developing grains is sensitive to growth conditions especially to the level of sulfate [43], [44], nitrogen [45] or day length [46]. It is therefore not surprising that hordein content differed between cultivars and locations. This observation adds an additional difficulty in choosing a suitable flour to act as a universal calibration standard.

### Response of ELISA Systems Sandwich Assay to Purified Hordein Standards

**Figure 11:**
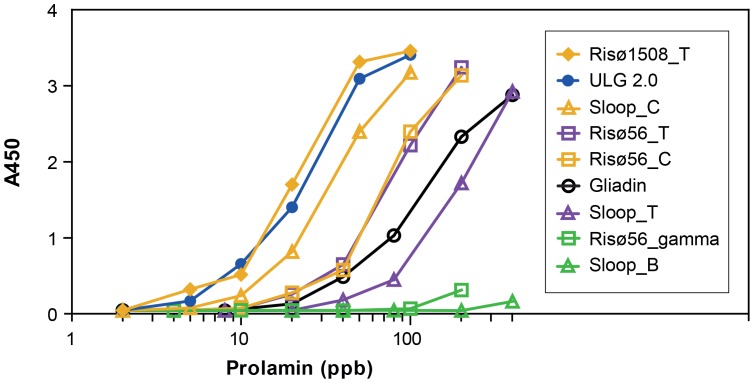
Response of *ELISA Systems* sandwich assay to log [hordein] concentration. The ELISA response to different hordein fractions, added in the concentrations indicated, are shown above in order of decreasing sensitivity.

Purified hordein standards were used to calibrate the response of ELISA kits. The response of the ELISA systems antibody to increasing hordein concentration may be illustrated for wide substrate concentrations by plotting on a log axes (Fig. 11). The curves of best fit approximate sigmoidal curves. However, when plotted on a linear scale, the curves of best fit accurately approximate standard Michaelis-Menton curves with individual R^2^ values >0.97. The sensitivity of the assay is described by the dissociation constant, Kd, analogous to the Km of a Michaelis-Menton curve. The Kd is a measure of the amount of hordein required to produce a half-maximal response in the ELISA assay (Table 1). The lower the Kd, the more sensitive was the detection, e.g. 57 ppb of ULG 2.0 total hordein, produced a half-maximal response in this assay, compared to 670 ppb Sloop total hordein required to produce the same colour (Fig. 11, Table 1). Thus ULG 2.0 hordein was detected approximately 10-fold more sensitively than total hordeins from Sloop. Thus the ELISA systems sandwich assay was very sensitive to ULG 2.0 and Risø 1508 total hordeins (Fig. 11, Table 1). Preparations enriched for B-hordeins (e.g. Table 1: Sloop_T, Sloop_B) were detected poorly. Likewise, the γ-hordein fraction from Risø 56 was also poorly detected, suggesting that the response to ULG 2.0 was due mainly to the D-hordein content as ULG 2.0 accumulates only D- and γ-3-hordein [33]. Preparations enriched for C-hordeins (e.g. Sloop_C, Risø 56_C, and Risø 56_T) were detected with medium sensitivity indicated by Kd values intermediate between those for total hordein from ULG 2.0 total and B-hordein (Table 1). One way analysis of variance (ANOVA) of log_10_ transformed Kd values confirmed each of these groups differed significantly (p<0.001; Table 1).

**Table 1:** Comparison of Kd of purified prolamins for *ELISA Systems* and *RidaScreen* sandwich assays.

Hordein^a^	*ELISA Systems* b		*RidaScreen* b	
	Kd±SE (ppb)	Log_10_ Mean Kd	Kd±SE (ppb)	Log_10_ Mean Kd
ULG 2.0_T	57±0.7	1.756^ #^	Not detected	
RisØ 1508_T	64±9.0	1.732^ #^	Not detected	
Sloop_C	84±0.7	1.924^ #∧^	15.8±0.3	1.198^g^
RisØ 56_C	212±24	2.320 ^∧*^	7.6±0.3	0.8745^g^
RisØ 56_T	261±58	2.393^ *†^	10.5±0.2	1.021^g^
Gliadin	343±6.0	2.535^ *†^	390±15	2.589^h^
Sloop_T	670±160	2.819^ †^	26±1.0	1.416^g^
RisØ 56_γ	3,640±180	3.560^ ‡^	560±90	2.697^h^
Sloop_B	19,400±3,600	4.272^ •^	2100±360	3.267^h^

The data were replotted as linear Michaelis-Menton plots, and the Kd values determined from the curves of best fit (GraphPAD Prism 5.04). Means±SE of two experiments are shown.

a The suffix _T refers to total hordein; _C refers to FPLC purified C-hordeins; _γ refers to FPLC purified γ-hordeins; _B refers to FPLC purified B-hordeins. Commercial gliadin standards used are denoted as Gliadin.

b The Kd values were log_10_ transformed to correct for heterogeneity of variance, and one-way ANOVA conducted on the data for each assay type. In both analyses the p-values for the F-tests for hordein were <0.001. Within each assay type log_10_(Kd) values with the same symbol were not significantly different at the 5% experiment-wise error rate (GenStat). A two-way ANOVA conducted on data for both assay types (excluding ULG 2.0_T and RisØ 1508_T hordeins) indicated a strong interaction between hordein and assay type (p<0.001), largely due to a lack of a significant difference between the gliadin response for each assay type (p = 0.734).

### Response of Ridascreen Sandwich ELISA to Purified Hordein Standards

**Figure 12:**
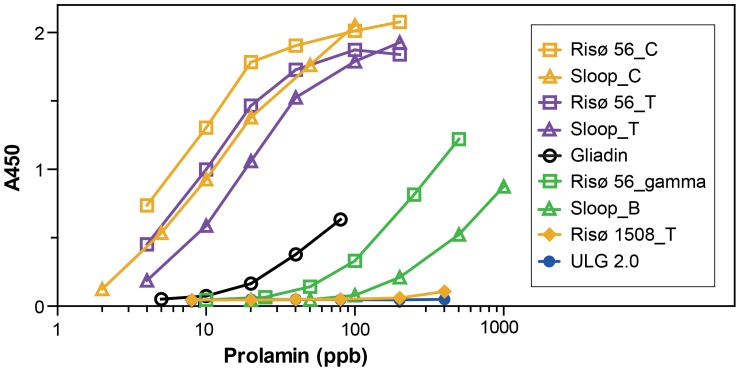
Response of *Ridascreen* sandwich assay to purified hordein standards. The ELISA response to different hordein fractions, added in the concentrations indicated, are shown above in order of decreasing sensitivity.

The *Ridascreen* antibody kit also bound to hordein proteins with varying affinity (Fig. 12, Table 1). The response of the *Ridascreen* kit to purified hordein fractions also accurately fitted sigmoidal curves when plotted on a log axis (Fig. 12). Kd values can be similarly extracted from linear plots. Not unexpectedly, the sensitivity of these antibodies to individual hordeins differs from that of the ELISA Systems antibodies. Thus the *Ridascreen* kit was relatively more sensitive to fractions enriched for C-hordeins, such as total hordeins from RisØ 56 and C-hordeins fractions purified from RisØ 56 or Sloop, having Kd values 15, 30 and 26 times lower (more sensitive) than the respective Kd values for the ELISA Systems kit (Table 1, Fig. 12). Preparations enriched for B-hordeins (e.g. Sloop_B) were detected with low sensitivity (Table 1). The γ-hordein fraction from Risø 56 was detected more sensitively by the *Ridascreen* kit, with a Kd value 6.5 times lower than that obtained with the ELISA Systems kit. However, hordein preparations enriched for D-hordeins, such as total hordein from ULG 2.0 and Risø 1508, were not detected at these dilutions by the *Ridascreen* kit (Fig. 12). Thus the *Ridascreen* kit was more sensitive to C-hordeins, but relatively insensitive to B-, D- and γ-hordeins. One way ANOVA analysis of log_10_ transformed Kd values for the *Ridascreen* kit showed there were two groups of hordeins, that containing the C-hordeins, and total hordeins from RisØ 56 and cv Sloop, which differed significantly from that containing gliadin, and B- and γ-hordeins (p<0.001; Table 1).

Both the ELISA Systems and *Ridascreen* kits have a similar sensitivity to gliadin with Kd values of 343 and 390 ppb respectively. Two way ANOVA (GenStat) showed the Kd values for gliadins did not vary significantly between antibody kits (p = 0.734; Table 1).

### Response of Both Sandwich ELISA Kits to Beer

**Figure 13:**
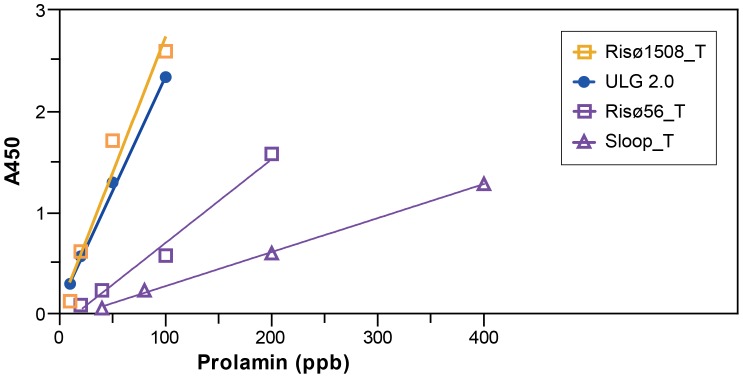
Response of *ELISA Systems* sandwich assay to purified total hordeins are shown.

At low concentrations of the total hordeins, the standard curves produced with the ELISA Systems kit approximate straight lines with individual R^2^ values >0.9 when plotted on a linear axis. The difference in sensitivity is reflected in the increased slopes of the ULG 2.0 and RisØ 1508 standard curves compared to the slope of the standard curves of the B-hordein rich lines, RisØ 56 and Sloop (Fig. 13). These standard curves correspond to total hordein preparations from the test grains and may be used to calculate accurately the hordein content of flour, and to approximate the hordein content of beers produced from these malted grains (Table 2).

**Table 2:** Comparison of hordein in flour and beer samples by *ELISA Systems* sandwich ELISA kit.

Line	*Elisa Systems* 1				
	Hordein in flour		Hordein in beer		Hordein in flour/hordein in beer
	mg/g flour	%	ppm	%	
Sloop	56.6±3.3	100	130±4.3	100	435
Risø 56	33.3±1.1	59	16.4±1.6	12.6	2,640
Risø 1508	4.9±0.3	8.7	0.82±0.00	0.6	8,170
ULG 2.0	1.7±0.1	3.0	12.7±0.10	9.7	134

1 The *ELISA Systems* data is reproduced from (Tanner et al, this volume, this journal) with permission.

Hordein in flour and beer were calibrated against an appropriate hordein standard prepared from the respective flour.

The response of ELISA Systems kit to beer was investigated by diluting the standard beers. As the dilution increased, the signal decreased as expected and the response was generally linear between A450 values of 0.5 and 1.5. Dilutions that produced an A450 in this linear range were compared to the appropriate standard curve (Fig. 13) and used to calculate the hordein concentration in each beer (Table 2). The response to standard beers was of a similar order as the respective Kd for total purified hordeins bearing in mind that the ULG 2.0 hordeins were approximately 10-fold more reactive than total Sloop hordeins (Table 1). Beer made from Sloop had the highest response at 130 ppm, followed by beer from RisØ 56, ULG 2.0, and RisØ 1508 at 16.4, 12.7 and 0.8 ppm respectively (Table 2). These values did not reflect the hordein content of the respective flours. The ratio of hordein in flour to hordein in respective beer varied from approximately 6,000 fold for Risø 1508, to a low of 134 fold for ULG 2.0 beer (Table 2). The proportion of flour hordein that dissolved in the final beer appeared to be related to the concentration in flour as well as the composition of the hordein.

## Discussion

The sensitivity of the sandwich ELISA kits tested here to purified hordeins varies considerably. Different kits show variation in the Kd for the same hordein standard e.g. total hordein from ULG 2.0 and RisØ 1508 were not detected by *Ridascreen*, but were sensitively detected by the *Elisa Systems* kit. The Kd of the different hordeins determined by *ELISA Systems* kit varied by 340-fold. The variation seen for the *Ridascreen* kit towards different hordeins was similar at 270-fold.

Determination of hordein by ELISA based methods report hordein concentrations in beverages in trace amounts, in the ppm range [13], [14], [15], [25]. It is likely that these levels are underestimates. In an accompanying paper we show that 20% of ELISA results for beers were false negatives compared to results obtained by relative mass spectrometry (Tanner et al, this journal, this issue). The generally accepted safe level for gluten is less than 20 ppm in food and drink by current methods [4]. The true hordein level is likely to be too high for safe consumption by coeliac, gluten intolerant or gluten allergic subjects, who are advised to avoid beer and malted products for life.

The ELISA Systems kit was sensitive to dilute beer, however analysis of the full Skerritt epitope PQPQPFPQE, against known hordeins reveals that it occurred in only one of the hordein families, C-hordeins (GeneBank accession AAA92333.1) and the alternate epitope PQQPPFPEE did not occur at all (Table S1). Since the Skerritt antibody detected other hordeins with adequate sensitivity (Table 1, Fig. 1C) it appears the binding is due to one or more partial epitopes present in the full PQPQPFPQE peptide.

The R5 epitope QQPFP occurs in a number of hordeins. For example the epitope appears in the B1-hordein (P06470) five times, the B3-hordein (P06471) twice, the B-hordeins (Q40026 and C7FB16) five times and one time respectively; the C-hordeins (Q40053, Q41210, and AAA92333.1) eight, sixteen and thirteen times respectively; γ-1-hordein (P17990) three times, the γ-3-hordein (P80198) once, but not in the D-hordein (Q84LE9) (Table S1). The R5 epitope QQQFP occurs once in γ-1-hordein (P17990) and three times in γ-3-hordein (P80198). The R5 epitope LQPFP occurs once in γ-1-hordein (P17990). The R5 epitope QLPFP occurs in only one hordein, C-hordein (AAA92333.1) once (Table S1).

However, the QQ epitope occurs in a variety of proteins, ranging from a low of 2, 1 and 1 mole QQ per mole protein for β-glucosidase, cystatin and LTP-2 respectively (Table 3). We suggest the QQ epitope may be responsible for the weak binding seen in these non-hordein proteins (Fig. 2). The QQ epitope occurs much more frequently in hordeins, ranging from 26 mole QQ per mole protein for γ-1-hordein to a high of 46 mole QQ per mole protein for C-hordeins.

**Table 3:** The ratio of moles of QQ/mole of protein calculated from molecular weight and sequence shown in Table S1.

Uniprot accessions	Protein	mole QQ/mole protein	MW	nmole QQ/ug protein
AAA92333.1^1^	C hordein	46	40,547	1.13
Q41210	C-hordein	40	36,508	1.10
P80198	γ-hordein-3	36	33,189	1.08
P06471	B3-hordein	32	30,195	1.06
P06470	B1-hordein	33	33,422	0.99
Q40053	C-hordein	29	30,396	0.95
Q40026	B-hordein	31	33,503	0.93
C7FB16	B-hordein	28	33,503	0.84
Q84LE9	D-Hordein	61	80,410	0.76
P17990	γ-hordein-1	26	34,737	0.75
P20145	Lipid transfer protein (LTP2)	1	10,357	0.10
Q9LEI7	Cystatin	1	11,780	0.08
Q40025	β-glucosidase	2	57,445	0.03

All accessions are Uniprot except 1: GenBank accession.

The results presented here show that quantification of trace hordeins levels by ELISA is problematic. The difficulty lies in obtaining a suitable standard to calibrate the method. Hordein standards extracted from appropriate flour samples may be used to calibrate assays to determine the hordein level in flour, or products that are not highly processed. However, this is at best a poor approximation for hordein in beer, since the hordein composition of the beer does not reflects the composition of the flour. As malting, mashing and brewing progress the protein profile changes in favour of heat-stable, water soluble proteins [47], [48]. Firstly, trace flour proteins may be dramatically enriched during brewing, e.g. serpin Z4 and LTP-1 and LTP-2. The dominant hordeins in flour become minor hordeins in beer, e.g. the C-hordeins [49]. The protein profile of flour, malt, wort, and beer by SDS-PAGE differ considerably. We show that flour hordeins can be reduced by up to 6,000 fold in beer. Such changes have previously been reported [14]. Secondly, proteins may be modified by hydrolysis, glycation or glycosylation during malting, mashing and brewing [47], [50], [51], [52]. Modification of an internal lysine by the addition of a sugar moiety may prevent the recognition of an amino-acid epitope by an antibody. For example, up to 16% of the lysine content of serpin Z4 is glycated during the brewing process [53]. Serpin Z4 may be used as a proxy for glycation of other proteins including hordeins, and it is likely that glycated lysine residues may mask at least some peptides from identification by selective anti-hordein antibodies. The glycosylation of gamma-3-hordein was recently shown to reduce epitope availability [54]. Glycation may also interfere with the detection and identification of peptides by MS, by interfering with the tryptic cleavage at either lysine or arginine, however, MS analysis to date has revealed only a single glycated residue in D-hordein (Tanner et al this journal, this issue).

The most likely way forward with the problem of quantification of gluten in food and beverages is the development of fully quantitative mass spectrometry based methods, to calculate the absolute concentration of hordein peptide, by spiking beer samples with several standard peptides, each representative of a hordein family and present in beer.

## Methods

### Plant Material

Barley line cv Sloop (wild type) was obtained from the Australian Winter Cereals Collection (Tamworth, Australia). Single hordein-null lines, Risø 56 (accumulating no B-hordeins due to a X-ray induced chromosomal deletion of a portion of chromosome 1H) and Risø 1508 (an ethyleneimine induced point mutation in the *lys 3a* gene on chromosome 5H which prevents accumulation of C-hordeins and decreased D- and B- hordeins [55], [56]) were obtained from the Nordic Germplasm Bank (Alnarp, Sweden), intercrossed and F2 progeny selected that lacked B- and C-hordeins. This hordein double-null seed was refined by single seed descent to produce an F6 line, Ultra Low Gluten barley (ULG 2.0). Plants were initially grown in glasshouse conditions at 25°C days and 20°C nights and harvested seeds inspected to exclude contamination. This stock of single and double hordein-null lines was increased in the field, harvested and malts and beers were prepared as described below.

### Preparation of Wort and Beer

For malting and brewing experiments cv Sloop, Risø 56 and Risø 1508 were grown side by side, at CSIRO Ginninderra Experiment Station, Canberra, in the field, and 10 kg of each harvested in December, 2007. The grains were malted by Barrett Burston Malting Co. Pty. Ltd., Richmond, Victoria, and 20 L batches of beer brewed by O’Brien Brewing, Ballarat, Victoria, using standard techniques. Optimum malting conditions were determined for each line. Barley was malted in a Joe White Micromalting System in several 800 g tins. The steeping regime involved: 8 h soaking, 9 h rest, 5 h soaking at 17°C (Sloop); 8 h soaking, 10 h rest, 5 h soaking at 17°C (Risø 56); and 7 h soaking, 8 h rest, 3 h soaking at 17°C (Risø 1508); 3 hrs soaking at 17°C (ULG 2.0). Germination occurred over 94 h at 16°C for Sloop and 15°C for the two hordein deletion mutants and for ULG 2.0 at 15°C for 96 hours. The kiln program was over 21 h between 50°C and 80°C. The kilned malt was mashed as detailed in Table 4. After the indicated amylase rest time, the mash was bought to the boil and boiled for 1 h to produce the wort. During boiling, the boiling wort was bittered with Tettnang hops to achieve 21–22 IBUs. The wort was cooled overnight to 20°C and then fermented with Fermentis US-05 yeast at 18–20°C to completion after about 2 weeks. The unfiltered beer was kegged, and force carbonated before bottling.

**Table 4:** Brewing protocols for standard beers.

	Sloop	Risø 1508	Risø 56	ULG 2.0
Volume (litres)	15.0	14.1	18.6	18.0
Malt (kg)	3.60	3.33	4.00	4.55
Protein rest(Temp/time)	57°C/20min	56°C/20 min	54°C/20 min	55°C/20 min
Amylase rest(Temp/time)	65°C/1 h	63–65°C/1 h	64–65°C/1 h	64–65°C/2 h
Original gravity (SG)	1.051	1.052	1.051	1.049
Final gravity (SG)	1.014	1.013	1.012	1.017
Alcohol (%vol)	4.8%	5.1%	5.2%	4.2%

This is was previously published in [49] and is reproduced here with permission for clarity.

### Protein Extraction

Total protein extracts were prepared by homogenising triplicate 20 mg samples of wholemeal flour or crushed triplicate endosperm halves of seeds, in 1 mL fresh, solvent containing 8 M urea, 1% (w/v) DTT, and 20 mM triethylamine-hydrochloride, all adjusted to pH 6 at 4°C (Urea/DTT), in a Bio101 bead beater (Savant) with 0.1 g of 0.1 mm dia. glass beads (Daintree Scientific, Tasmania), a ¼ inch ceramic bead (Bio101 systems, MP Biomedicals, California) for 30 s at speed 4, taking care not to overheat the solution. Extracts were centrifuged at 13,000 g av.

Alcohol soluble extracts were prepared by homogenising triplicate 20 mg samples of wholemeal flour, or crushed triplicate endosperm halves of seeds in 1 mL of 0.5 M NaCl and centrifuging at 10,000g for 10 min. This process was repeated twice more and the pellet finally extracted with 0.5 mL of 50% (v/v) isopropyl alcohol (IPA), containing 1% (w/v) DTT (IPA/DTT) at RT and centrifuged.

The distribution of protein between different solubility fractions was carried out by first extracting triplicate 20 mg samples of wholemeal flour or finely ground malt in 1 mL of aqueous buffer containing 20 mM TEA-HCl (pH 8.0), 1% (w/v) sodium ascorbate, 1% (w/v) PEG 6000, and 1 µg/mL of the protease inhibitors AEBSF and E64, centrifuged and repeated three times. PEG was added to prevent proteins binding to testa/seed coat proanthocyanidins present in barley. Ascorbate minimises the self-catalytic formation of quinone free radicals from proanthocyanidins metabolites, which would otherwise covalently bind to proteins [57]. The pooled supernatants were the water soluble protein fraction. The pellet was extracted three times with 0.5 M NaCl as above. The supernatant was the salt soluble fraction. The pellet was extracted in IPA/DTT three times as above, and the supernatant was the alcohol soluble fraction. The pellet was then extracted three times in Urea/DTT as above and the pooled supernatants were the urea soluble fraction.

### One Dimensional SDS-polyacrylamide Gel Electrophoresis (SDS-PAGE)

Up to 10 µL of alcohol soluble or total protein solution was diluted to a final volume of 30 µL with a solution containing 8 M urea, 2% (w/v) SDS, 62.5 mM Tris-HCl (pH 6.8), 0.01% (w/v) bromophenol blue containing 65 mM fresh DTT (Urea/SDS), left to reduce at RT for 30 min, applied to NuPage 4–12% Bis-Tris acrylamide gel (Invitrogen), calibrated with 10 kDa protein ladder (Invitrogen) and electrophoresed at 200 V for 60 min. Gels were fixed in 40% (v/v) MeOH, 10% (v/v) acetic acid, washed in distilled water, and proteins stained in 0.006% (w/v) colloidal Commassie G250, conveniently made by dissolving 150 mg Commassie G250 (Bio-Rad) in 125 mL EtOH, filtering with constant stirring into a final volume of 2.5 L MilliQ water containing 250 mL of 85% (w/v) phosphoric acid, and destained in water overnight.

### Western Blotting

SDS-PAGE was carried out as above and protein gels were blotted without delay to a nitrocellulose membrane at 20 V for 7 min (iBlot Invitrogen) and the membrane stained in a solution containing 0.2% (w/v) Ponceau-S (Sigma), 3% (w/v) trichloroacetic acid, and 3% (w/v) 5-sulphosalicylic acid. The image was scanned on an Image Scanner III (GE Healthcare) using Labscan software (GE Healthcare), the membrane destained in water, and blocked in 5% (w/v) skim milk powder in 1% (w/v) Tween in phosphate buffered saline (PBST) for 2 h at RT.

Blots were firstly interrogated with commercial polyclonal anti-gliadin-HRP antibody conjugate (Sigma), at 1/2000 dilution in PBST, developed in ECL reagent (Enhanced chemiluminescence, GE Health Care), and exposed to Hyperfilm (GE Health Care). Where indicated membranes were then stripped, re-blocked and probed with a second antibody pair: rabbit polyclonal anti-hordein at 1/2000 for 1 h followed by 1/2000 diluted donkey anti-rabbit-HRP (GE Healthcare) for 30 min and developed as above. This procedure was repeated for the third antibody pair: mouse monoclonal anti-gliadin (MAb 12224, Skerrit [10]), followed by 1/2000 diluted donkey anti-mouse-HRP (GE Healthcare). Stripped membranes were checked for lack of residual signal before applying the next antibody.

### Extraction and Purification of Hordein Standards

Water soluble proteins were extracted from 10 g fine wholemeal flour by washing three times, with 120 ml aqueous buffer containing 20 mM TEA-HCl (pH 8.0), 1% (w/v) sodium ascorbate, 1% (w/v) PEG 6000, and 1 µg/mL of the protease inhibitors AEBSF and E64. Alcohol soluble proteins in the pellet were dissolved by stirring in 40 mL of 50% (v/v) isopropyl alcohol (IPA) containing 1% (w/v) DTT (IPA/DTT), precipitated with 2 volumes of IPA at −20°C over-night. SDS-PAGE showed all hordeins were precipitated by this procedure. Precipitated hordeins were collected by centrifugation and dissolved in 10 mL of 8M urea, 1% (w/v) DTT and 20 mM TEA-HCl, all adjusted to pH 6 at 4°C (Urea/DTT) and purified by repeated injections of 1 mL Urea/DTT solution onto an 8 mL Source reverse phase FPLC column (GE Health Sciences) eluted with a linear gradient from 5 to 30% solvent B (60% (v/v) aqueous acetonitrile, 0.1% (v/v) triflouroacetic acid (TFA ) over 2.5 mL, and 30% B to 85% B over 70 mL at 4 mL/min (Fig. 1). The column was then washed with 100% B and re-equilibrated in 5% B. Solvent A was aqueous 0.1% (v/v) (TFA). Total hordein fractions were isolated after re-chromatography and lyophilisation of pooled FPLC eluates from 30 to 80 mL as indicated (Fig. 1A) from cv Sloop, Risø 56, Risø 1508 and ULG 2.0. Fractions enriched for B-hordeins were isolated as indicated from Sloop (Fig. 1B, peak 4); C-hordeins from Risø 56 (Fig 1C, peak 8), and Sloop (Fig 1B, peak 3); and γ-hordeins from Risø 56 (Fig. 1C, peak 9) and re-chromatographed and lyophilised. It has not been possible to purify a D-hordein fraction. Primary hordein standards were prepared by dissolving the lyophilised hordein fractions at 1.0 mg/mL in a solvent containing 8 M urea, 1% (w/v) DTT and 20 mM triethylamine-HCl, adjusted to pH 6 at 4°C (Urea/DTT) and stored at −20°C for up to 6 months. Aliquots were freeze/thawed no more than five times without loss of ELISA response (Fig. 9) or change in SDS-PAGE patterns. The composition of these fractions was verified by FPLC (Fig. 5), SDS-PAGE (Fig. 6) and protein in-gel digestion and peptide sequencing [49].

### ELISA Analysis

The response to purified hordein standards was measured with *RidaScreen* (#R7001, R-Biopharm AG, Darmstadt) or ELISA Systems (#ESGLI-48, *ELISA Systems*, Windsor, Queensland) sandwich ELISA assay kits, with variation to reaction times and temperatures as below. The hordein content of beer was determined using the ELISA Systems kit.

### ELISA Determination (ELISA Systems Kit)

The primary hordein standards were thawed and diluted in ELISA Systems sample diluent containing 0.2 mM excess H_2_O_2_ (ED buffer) to give: 400 parts per billion (ppb, 40 ng hordein/100 µL) for gliadin, cv Sloop total hordein and B-hordein from cv Sloop; 200 ppb for total hordein and C-hordein from Risø 56 or γ-hordein from Risø 56; or 100 ppb of C-hordein from cv Sloop or total hordein from ULG 2.0 and Risø 56. Further serial dilutions as indicated (Fig. 11) produced a standard curve for each prolamin in a final volume of 100 µL in the ELISA wells. These solutions were incubated for 1 h at 37°C to bind prolamins. Unbound protein was removed with 3 washes of PBST, then 100 µL of antibody-peroxidase conjugate was added per well and incubated at 37°C for 30 min, washed three times with PBST. Finally 100 µL of substrate solution was added and the reaction stopped after 15 min at 37°C with 1 M phosphoric acid and the A450 determined (Multiscan, Thermo Vantaa, Finland).

Alcohol soluble protein extracts were isolated from aqueous washed flour, dissolved in IPA/DTT and the protein content measured. The hordein content was determined by adding 40 ng total protein in a final volume 100 µL ED buffer. The ELISA wells contained a maximum of 16 mM urea and 0.13 mM DTT and were treated as above, and calibrated against a 0.2 mM excess H_2_O_2_ quenched standard curve of the appropriate total hordein: Sloop total hordein for Sloop flour, Risø 56 total hordein for Risø 56 flour, Risø 1508 total hordein for Risø 1508 flour, or ULG 2.0 total hordein for determination of hordein in ULG 2.0 flour (Table 2).

Hordein was determined in degassed test beers brewed from Sloop, Risø 56, Risø 56 and ULG 2.0 diluted with ED buffer so that the response was in the linear portion of the appropriate standard curve. Duplicate aliquots of suitably dilute beer were added directly to ELISA wells, treated as above, and calibrated against a 0.2 mM excess H_2_O_2_ quenched standard curve of appropriate hordeins as above (Table 2).

### Effect of Container on ELISA Determination (ELISA Systems Kit)

The effect of following containers on the ELISA Systems kit were examined: Axygen Biosciences 1.7 mL microtubes (Union City, California, USA), Greiner BioOne Cellstar 15 mL tubes (Frickenhausen, Germany), National Scientific Supply Co. 1.1 mL microtubes (Claremont, California, USA) or Greiner BioOne 96×1 mL Master Block (Frickenhausen, Germany).

### ELISA Determination (RidaScreen Kit)

The primary hordein standards were thawed and diluted in *Ridascreen* sample diluent (RD buffer) to give final concentrations of: 400 ppb for cv Sloop total hordein or B-hordeins from cv Sloop; 200 ppb for total hordeins or C- and γ-hordeins isolated from Risø 56; 100 ppb for C-hordeins from cv Sloop or total hordeins from Risø 56 and ULG 2.0; or 80 ppb *Ridascreen* gliadin standard, and added to sample wells. Further serial dilution as indicated (Fig. 12) produced a standard curve for each prolamin in a final volume of 100 µL in the ELISA wells. These solutions were incubated for 1 h at 37°C to bind prolamins. Unbound protein was removed with 3 washes of PBST, then 100 µL of 1/11 diluted antibody-enzyme conjugate added, incubated at 37°C for 30 min, washed three times with PBST. Finally 100 µL (1∶1 v/v) substrate/chromogen solution was added and the reaction stopped after 15 min at 37°C with 1 M sulfuric acid and the A450 determined (Multiscan, Thermo Vantaa, Finland).

### Statistical Analysis

Mean ELISA readings were transformed as needed to achieve homogenous variance, via either log10(1+concentration) or log10(concentration) as discussed in legends of Table 1, and Figures 8, 9, and 10. In each case significant differences were determined using a one way analysis of variance of the transformed means (ANOVA; GenStat [58]).

Protein concentration was determined by the method of Bradford [59].

## Supporting Information

Table S1 (DOC)
