# Supplementary material for: Quantification of Hordeins by ELISA: The Correct Standard Makes a Magnitude of Difference
Source: PLoS One. 2013 Feb 28;8(2):e56456. doi: 10.1371/journal.pone.0056456 (PMC3585327; doi:10.1371/journal.pone.0056456)
Supplement: Table S1 — (DOC) [file pone.0056456.s001.doc]

**Supplementary Table:**

**Table S1:**

**HORDEINS**

>sp|P06470|HOR1_HORVU B1-hordein OS=Hordeum vulgare PE=1 SV=1

MKTFLIFALLAIAATSTIA**QQQPFPQQ**PIP**QQ**PQPYP**QQ**PQPYP**QQPFP**P**QQPFPQQ**PVP**QQ**PQPYP**QQPFP**P**QQPFPQQ**PPFW**QQ**KPFP**QQ**PPFGL**QQ**PILS**QQ**QPCTP**QQ**TPLPQGQLYQTLLQLQIQYVHPSIL**QQ**LNPCKVFL**QQ**QCSPVPVPQRIARSQML**QQ**SSCHVL**QQ**QCCQQLPQIPEQFRHEAIRAIVYSIFLQEQP**QQ**LVEGVSQP**QQ**QLWP**QQ**VGQCSF**QQ**PQP**QQ**VG**QQQQ**VPQSAFLQPHQIAQLEATTSIALRTLPMMCSVNVPLYRILRGVGPSVGV

Theoretical pI/Mw: 8.86 / 33422.45

>sp|P06471|HOR3_HORVU B3-hordein (Fragment) OS=Hordeum vulgare PE=1 SV=1

**QQ**PVSRQP**QQ**IIP**QQ**P**QQPFP**LQP**QQ**PQPFP**QQ**PIP**QQ**PQPYP**QQ**PQSFP**QQPFP**S**QQ**PFP**QQ**PPFW**QQ**QPVLS**QQ**QPCTQDQTPLLQE**QQ**DQMLVQVQIPFVHPSIL**QQ**LNPCKVFL**QQ**QCSPLAMSQRIARSQML**QQ**SSCHVL**QQ**QCC**QQ**LPQIPEQLRHEAVRAIVYSIVLQEQSLQLVQGVSQP**QQ**QS**QQQQ**VGQCSF**QQ**PQP**QQ**G**QQQQ**VPQSVFLQPHQIAQLEATTSIALRTLPTMCSVNVPLYRIVPLAIDTRVGV

Theoretical pI/Mw: 7.74 / 30195.42

>tr|Q40026|Q40026_HORVU B hordein OS=Hordeum vulgare PE=4 SV=1

MKTFLIFALLVIAATSTIA**QQQPFPQQPFPQQ**PQPYP**QQ**PQPYP**QQ**PFQP**QQPFPQQ**TIP**QQ**PQPYP**QQPFP**P**QQ**EFP**QQ**PPFWP**QQPFPQQ**PPFGL**QQ**PILS**QQ**QPCTP**QQ**TPLPQGQLYQTLLQLQIPYVHPSIL**QQ**LNPCKVFL**QQ**QCSPVRMPQLIARLQML**QQ**SSCHVL**QQ**QCCQQLPQISEQFRHEAIRAIVYSIFLQEQP**QQ**SVQGVSQT**QQ**QL**QQ**EQVGQCSF**QQ**PQP**QQ**LGQA**QQ**VPQSVFLQPHQIAQLEATTSIALRTLPRMCNVNVPLYDIMPPDFWH

Theoretical pI/Mw: 6.93 / 33503.36

>tr|C7FB16|C7FB16_HORVD B hordein OS=Hordeum vulgare var. distichum PE=4 SV=1

MKTFLIFALLAIVATSTIA**QQ**QPYP**QQ**PQPFP**QQ**PIP**QQ**PQPYP**QQQQPFP**QRPFPS**QQ**PFP**QQ**PPFW**QQ**QPILSK**QQ**PCTP**QQ**PPLPQG**QQ**DQMLVQVQIPFVHPSIL**QQ**LNPCKVFLQ**QQ**CSPVAMSQRIARSQML**QQ**SSCHVL**QQ**QCC**QQ**LPQIPEQFRHEAVRAIVYSIVLQEQPQQLVQGVSQP**QQ**QSQL**QQ**VGQCSF**QQ**PQP**QQ**GK**QQ**QVPHSVFLQPHQLAQLEATASIALRTLPMMCSVNLPLYRILPFGIDTRVGV

Theoretical pI/Mw: 6.93 / 33503.36

>tr|Q40053|Q40053_HORVU Hor1-17 C-hordein OS=Hordeum vulgare PE=4 SV=1

MKTFLTFVLLAMVMSIVTTARQLNPSSQELQSP**QQ**SYL**QQ**PYPQNPYLPQKPFPV**QQ**PFHTP**QQ**YFPYLPEELFPQYQIPTPLQP**QQPFPQQ**P**QQ**PLPRP**QQPFP**WQP**QQPFP**QPQEPIP**QQ**P**QQPFPQQ**P**QQPFPQQ**P**QQ**IIF**QQ**P**QQ**SYPVQP**QQPFP**QPQPVP**QQ**RP**QQ**ASPLQPQP**QQ**ASPLQP**QQPFP**QGSEQIIP**QQPFP**LQPQPFP**QQ**P**QQ**PLPQP**QQ**PFR**QQ**AELIIP**QQ**PQQPLPLQPHQPYT**QQ**TIWSMV

Theoretical pI/Mw: 6.71 / 30396.51

>tr|Q41210|Q41210_HORVU C-hordein OS=Hordeum vulgare GN=C-hordein PE=4 SV=1

MKTFLTFVLLAMVMSIVTTARQLNPSSQELQSP**QQ**SYL**QQ**PYPQNPYLP**QQPFP**V**QQ**PFHTP**QQ**YFPYLPEELSPQYQIPTPLQP**QQPFPQQ**P**QQ**PLPRP**QQPFP**WQP**QQPFP**QP**QQ**PIPYQP**QQ**PFN**QQ**P**QQ**IIS**QQ**P**QQPFPQQ**P**QQPFP**QP**QQPFP**WQP**QQPFP**QP**QQPFP**LQP**QQ**PFPWQP**QQPFP**QP**QQ**PIAHQP**QQ**PFSFS**QQ**P**QQPFP**LQP**QQPFPQQ**P**QQPFPQQ**P**QQ**IIFQQP**QQ**SYPVQP**QQPFP**QPQPVP**QQ**RP**QQ**ASPLQP**QQPFP**QGSEQIIP**QQ**P**QQPFP**LQPHQPYT**QQ**TIWSMV

Theoretical pI/Mw: 6.81 / 36508.14

>gi|893242|gb|AAA92333.1| C hordein [Hordeum vulgare subsp. vulgare]

MKTFLTFVLLAMAMSIVTTARQLNPSHQELQSPQQPFLKQQSYLQQPYPQQPYLP**QQPFP**TPQQFFPYLPQQTFPPS**QQ**PNPLQP**QQPFP**LQPQPP**QQPFP**QP**QQ**PNP**QQ**P**QQPFP**RQP**QQ**IVP**QQ**P**QQPFPQQ**P**QQPFP**QP**QQ**PFSWQP**QQ**PFLQPLQLXPLQA**QQPFP**LQPQLPFPQP**QQ**PIG**QQ**PKQPLL**QQ**P**QQ**TIP**QQ**P**QQPFP**LQP**QQPFPQQ**P**QQ**PLP**QQ**P**QQ**IIS**QQ**P**QQPFP**LQP**QQPFP**QPQPFPQEQP**QQ**AFPLQP**QQPFP**EESEQIIT**QQPFP**LQP**QQ**LFP**QQ**P**QQ**PLPQP**QQ**PFRQLPKYIIP**QQ**P**QQ**PFLLQPHQP**QQ**PYA**QQ**DIWSDIALLG

Theoretical pI/Mw: 6.71 / 40547.27

>tr|Q84LE9|Q84LE9_HORVU D-Hordein OS=Hordeum vulgare PE=4 SV=1

MAKRLVLFVAVIVALVALTTAEREINGNNIFLDSRSRQLQCERELQESSLEACRRVVD**QQ**LVGQLPWSTGLQMQCC**QQ**LRDVSPECRPVALSQVVRQYE**QQ**TEVPSKGGSFYPGGTAPPL**QQ**GGWWGTSVKWYYPDQTSS**QQ**SWQG**QQ**GYHQSVTSS**QQ**PGQG**QQ**GSYPGSTFP**QQ**PGQG**QQ**PGQRQPWSYPSATFP**QQ**PGQGQG**QQ**GYYPGATSLLQPGQG**QQ**GPYQSATSP**QQ**PGQGQG**QQ**EPYPIATSPHQPGQW**QQ**PGQG**QQ**GYYPSVTSP**QQ**SGQG**QQ**GYPSTTSP**QQ**SGQG**QQ**LGQG**QQ**PGQG**QQ**GYPSATFP**QQ**PGQW**QQ**GSYPSTTSP**QQ**SGQG**QQ**GYNPSGTST**QQ**PGQVQQLGQG**QQ**GYYPIATSP**QQ**PGQG**QQ**LGQG**QQ**PGHG**QQ**LVQG**QQ**QGQG**QQ**GHYPSMTSPHQTGQGQKGYYPSAISP**QQ**SGQG**QQ**GYQPSGASSQGSVQGACQHSTSSP**QQ**QAQGCQASSPKQGLGSLYYPSGAYT**QQ**KPGQGYNPGGTSPLH**QQ**GGGFGGGLTTEQPQGGKQPFHC**QQ**TTVSPHQG**QQ**TTVSPHQG**QQ**TTVSPHQG**QQ**TTVSPHQG**QQ**TTVSPHQG**QQ**TTVSPHQG**QQ**TTVSPHPG**QQ**TTVSPHQG**QQ**TTVSPHPG**QQ**TTVSPHQG**QQ**TTVSPHQG**QQ**TTVSPHQG**QQ**TTVSPHQG**QQ**TTVSPHQG**QQ**TTVSPHQG**QQ**PGEQPCGFPG**QQ**TTVSLHHG**QQ**SNELYYGSPYHVSVEQPSASLKVAKA**QQ**LAAQLPAMCRLEGGGGLLASQ

Theoretical pI/Mw: 8.01 / 80409.65

>sp|P17990|HOG1_HORVU Gamma-hordein-1 OS=Hordeum vulgare PE=2 SV=1

MKILIILTILAMATTFATSEMQVNPSVQVQPT**QQ**QPYPES**QQ**PFISQS**QQ**QFPQP**QQPFPQQ**P**QQPFP**QS**QQ**QCL**QQ**PQHQFPQPT**QQ**FPQRPLLPFTHPFLTFPDQLLPQPPHQSFPQPPQSYPQPP**LQPFP**QPP**QQ**KYPEQP**QQPFP**W**QQ**PTIQLYL**QQ**QLNPCKEFLL**QQ**CRPVSLLSYIWSKIV**QQ**SSCRVM**QQ**QCCLQLAQIPEQYKCTAIDSIVHAIFM**QQ**GQRQGVQIV**QQ**QPQP**QQ**VGQCVLVQGQGVVQP**QQ**LAQMEAIRTLVLQSVPSMCNFNVPPNCSTIKAPFVGVVTGVGGQ

Theoretical pI/Mw: 8.34 / 34736.94

>sp|P80198|HOG3_HORVU Gamma-hordein-3 OS=Hordeum vulgare PE=1 SV=1

ITTTTMQFNPSGLELERP**QQ**LFPQWQPLP**QQ**PPFL**QQ**EPEQPYP**QQ**QPLP**QQQPFPQQ**PQLPHQHQFP**QQ**LP**QQ**QFP**QQ**MPLQP**QQ**QFP**QQ**MPLQP**QQ**QPQFP**QQ**KPFGQY**QQ**PLT**QQ**PYP**QQ**QPLA**QQ**QPSIEEQHQLNLCKEFLL**QQ**CTLDEKVPLLQSVISFLRPHIS**QQ**NSCQLKR**QQ**CC**QQ**LANINEQSRCPAIQTIVHAIVM**QQ**QV**QQ**QVGHGFVQSQL**QQ**LGQGMPIQL**QQ**QPGQAFVLP**QQ**QAQFKVVGSLVIQTLPMLCNVHVPPYCSPFGSMATGSGGQ

Theoretical pI/Mw: 6.70 / 33188.80

**NON-HORDEINS**

sp|Q9LEI7|CYT1_HORVU Cysteine proteinase inhibitor (Cystatin) OS=Hordeum vulgare GN=ICY PE=1 SV=1

MAEAAHGGGLRGRGVLLGGVQDAPAGRENDLETIELARFAVAEHNAKANALLEFEKLVKVR**QQ**VVAGCMHYFTIEVKEGGAKKLYEAKVWEKAWENFKQLQEFKPAA

Theoretical pI/Mw: 6.84 / 11780.51

tr|Q40025|Q40025_HORVU Beta-glucosidase OS=Hordeum vulgare GN=BGQ60 PE=3 SV=1

MRSSPVLLLVIALVAAAHLAPLECDGPNPNPEIGNTGGLSRQGFPAGFVFGTAASAYQVEGMARQGGRGPCIWDAFVAIQGMIAGNGTADVTVDEYHRYKEDVGIMKNMGFDAYRFSISWSRIFPDGTGKVNQEGVDYYNRLIDYML**QQ**GITPYANLYHYDLPLALH**QQ**YLGWLSPKIVGAFADYAEFCFKVFGDRVKNWFTFNEPRVVAALGYDNGFHAPGRCSKCPAGGDSRTEPYIVTHNIILSHAAAVQRYREKYQPHQKGRIGILLDFVWYEPHSDTDADQAAAQRARDFHIGWFLDPITNGRYPSSMLKIVGNRLPGFSADESRMVKGSIDYVGINQYTSYYMKDPGAWNQTPVSYQDDWHVGFVYERNGVPIGPRANSDWLYIVPWGMNKAVTYVKERYGNPTMILSENGMDQPGNVSIADGVHDTVRIRYYRDYITELKKAIDNGARVAGYFAWSLLDNFEWRLGYTARFGIVYVDFNTLKRYPKDSALWFKNMLSEKKRS

Theoretical pI/Mw: 7.18 / 57444.95

SP|P20145|NLTP2_HORVU Probable non-specific lipid-transfer protein OS=Hordeum vulgare GN=LTP2 PE=2 SV=1

MAMAMGMAMRKEAAVAVMMVMVVTLAAGADAGAGAACEPAQLAVCASAILGGTKPSGECCGNLRA**QQ**GCLCQYVKDPNYGHYVSSPHARDTLNLCGIPVPHC

Theoretical pI/Mw: 6.78 / 10357.17
